# Urban emergence of *Dermanyssus gallinae* lineage L1 and *Ornithonyssus sylviarum* in Hungary: phylogenetic differentiation between the roles of migrating *vs* transported synanthropic birds

**DOI:** 10.1186/s13071-021-04643-3

**Published:** 2021-03-08

**Authors:** Sándor Hornok, Nóra Takács, Gábor Sipos, Pál Morandini, Attila D. Sándor, Sándor Szekeres, Andrea Grima, Jenő Kontschán

**Affiliations:** 1 Department of Parasitology and Zoology, University of Veterinary Medicine, Budapest, Hungary; 2 Hungarian Ornithological and Nature Conservation Society, 1125 Budapest, Hungary; 3 Department of Parasitology and Parasitic Diseases, University of Agricultural Sciences and Veterinary Medicine, Cluj-Napoca, Romania; 4 APH Veterinary Hospital, Attard, Malta; 5 Plant Protection Institute, Centre for Agricultural Research, ELKH, Budapest, Hungary

**Keywords:** Dermanyssoidea, Chicken red mite, Northern fowl mite, *Cox*1, 28S rRNA

## Abstract

**Background:**

Among Dermanyssoidea, the chicken red mite (*Dermanyssus gallinae*) and the northern fowl mite (*Ornithonyssus sylviarum*) are considered to be the cause of high economic losses endured by the poultry industry in the Holarctic region, with *O. sylviarum* predominating in North America and *D. gallinae* in Europe. Both species have a short life-cycle (thereby allowing a rapid build-up of massive infestations), a wide range of hosts, synanthropic presence and the ability to bite humans. The aim of this study was to analyze dermanyssoid mite specimens, collected in two human dwellings and two racing pigeon premises in different urban areas in Hungary, with molecular–phylogenetic methods.

**Methods:**

Mite species were identified morphologically. This was followed by DNA extraction and molecular–phylogenetic analyses of selected mites, based on the cytochrome *c* oxidase subunit I (*cox*1) and 28S rRNA (*28S*) genes.

**Results:**

Mites that had invaded a home from a pigeon nest and were linked to human dermatitis were morphologically and molecularly identified as *D. gallinae* special lineage L1. Specimens collected at all other sampling sites were identified as *O. sylviarum*, including mites that had invaded a home from a house martin (*Delichon urbicum*) nest, as well as those which were collected from racing pigeons. House martin- or pigeon-associated *O. sylviarum* specimens showed the highest sequence identity and closest phylogenetic relationship with conspecific mites reported in GenBank from Israel or Canada, respectively.

**Conclusions:**

Detailed morphological and molecular–phylogenetic analyses of *D. gallinae* lineage L1 confirmed its status as a cryptic species within *D. gallinae *(*s.l.*). Taking into account the well-documented latitudinal migratory routes of house martins between Hungary and Africa, *O. sylviarum* associated with this bird species most likely arrived on its host from the eastern Mediterranean region. On the other hand, mites collected from pigeons in Hungary showed *cox*1 genetic homogeneity with North American *O. sylviarum*, which can only be explained by a long-distance (west-to-east intercontinental) connection of birds and their mites as part of human activity (e.g. transportation to exhibitions or trading). In summary, this is the first molecularly confirmed and phylogenetically analyzed case of *O. sylviarum* infestation of birds in Hungary, implicating urban environment and involving distant parts of the country. This is also the first report of *D. gallinae* lineage L1 in central Europe. 
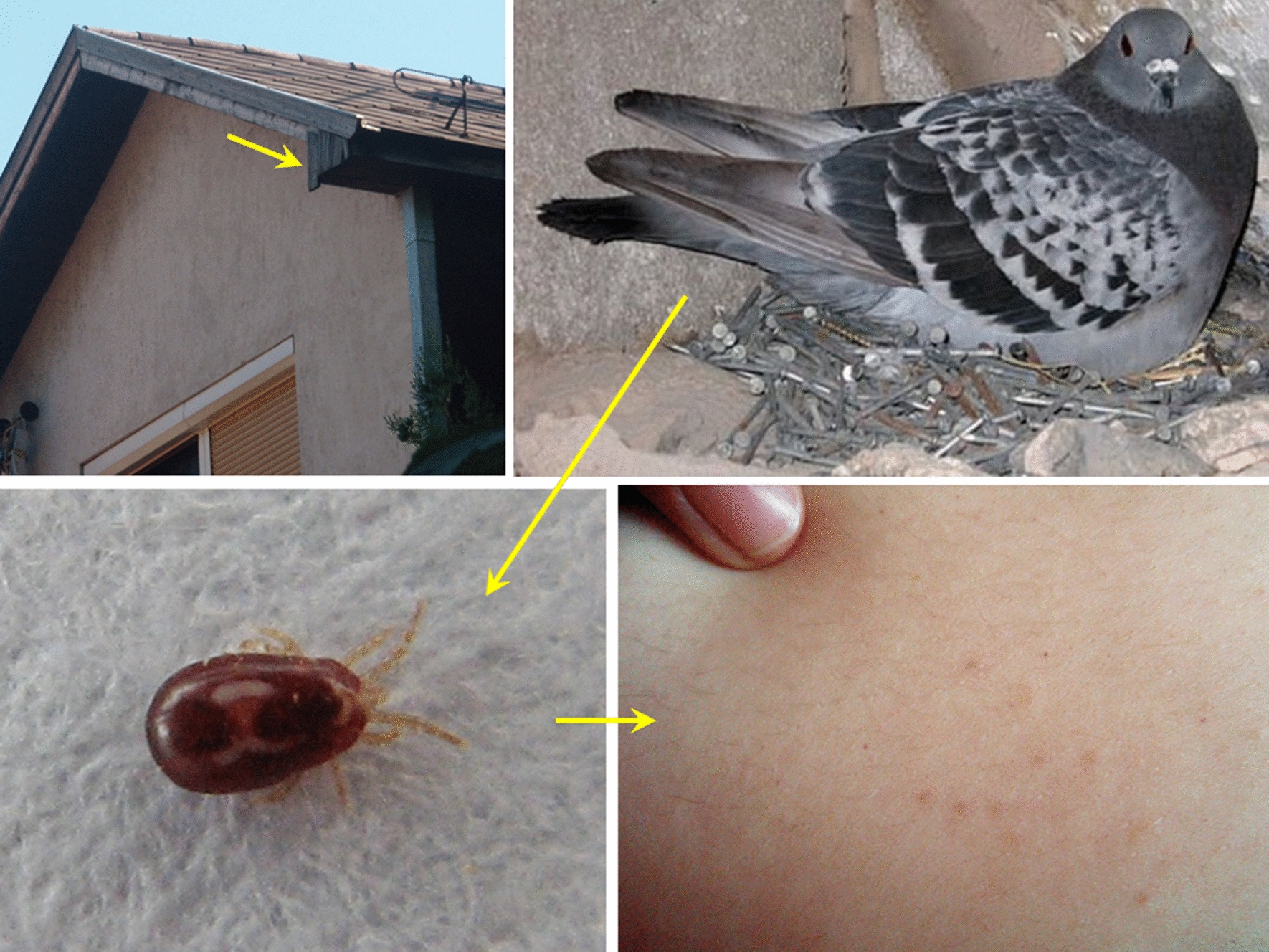

## Background

The Dermanyssoidea (Acari: Parasitiformes: Mesostigmata) is an extremely diverse lineage of mites that are found as free-living predators as well as facultative or obligate parasites of mammals, birds, reptiles and various arthropod groups [1]. Among the dermanyssoid mites, members of the families Dermanyssidae and Macronyssidae may have the highest veterinary-medical significance due to the hematophagous, obligatory ectoparasites in these families that affect animal husbandry and may also blood-feed on humans [2]. Adding to the nuisance and dermatitis they may cause, representatives of these two families may also transmit pathogens (viruses, bacteria as well as parasites) of importance to public and animal health [2–4]. In particular, the chicken red mite, *Dermanyssus gallinae* (family Dermanyssidae), and the northern fowl mite, *Ornithonyssus sylviarum* (family Macronyssidae), are responsible for high economic losses [5, 6] in the poultry industry and for threats to public health [3, 7].

In geographical terms, these two mite species appear to have a separate impact on the poultry industry, with *O. sylviarum* regarded as the most important mite pest in poultry farms in North America [6] and *D. gallinae* playing a similar role in Europe [8]. However, the risk of *O. sylviarum* being disseminated throughout the poultry industry in Europe has increased over the years, with several countries, including France [9], Sweden [10], Bulgaria [11] and Slovakia [12], reporting the emergence/presence of *O. sylviarum*, mostly on wild birds but also on poultry in some countries [8, 10]. Both *D. gallinae* and *O. sylviarum* are characterized by a very short life-cycle (under ideal conditions approximately 1 week [6, 7]), allowing a rapid build-up of local populations [8]. However, they differ in that *D. gallinae* is a nidicolous, temporary nocturnal ectoparasite [5] that tends to complete its blood meal quickly at the first opportunity [13] while *O. sylviarum* is a permanent ectoparasite that completes its entire life-cycle on its host [8], thus allowing partial and repeated feeding [13].

Despite its long-term attachment to its host, *O. sylviarum* is able to move short distances off-host, during which time it can survive for several weeks [8]. In contrast, *D. gallinae* can endure starvation for up to 8.5 months in the environment, where it moves rapidly, with speeds of up to 120 m/h [14]. This mechanism is important as it allows these mites to invade new environments (including those of humans) and other hosts locally. Both *D. gallinae* and *O. sylviarum* may parasitize a broad range of such hosts, most commonly birds (from multiple avian orders), but occasionally also mammals [15]. Among the latter, both mite species can bite humans [3, 7], causing localized pruritic, erythematous papular dermatitis. In artificial settings, *D. gallinae* is capable of starting blood-feeding on humans within 3 h [13], unlike *O. sylviarum*.

Knowledge of the life-cycle, host preference and synanthropic presence of these two dermanyssoid mites raises the question of whether long-distance natural bird migration and/or human activity (animal trade, transportation) may be responsible for the local introduction of *D. gallinae* or *O. sylviarum*. Although both species are significant ectoparasites of pigeons (order Columbiformes) [14] and migratory songbirds (order Passeriformes) [15], based on genetic evidence, animal transportation (and not bird migration) is thought to be responsible for the dispersal of *D. gallinae* in Europe [16]. This is essentially interrelated with its temporary parasitic nature. However, it is reasonable to hypothesize that long-distant dispersal and synanthropic arrival should be highly relevant in the case of the permanently parasitic *O. sylviarum*. This hypothesis has not yet been fully explored using phylogenetic methods.

In Hungary, *D. gallinae* has only been reported from poultry. In one study, four farms were examined, all of which had mite problems despite frequent chemical control [17]. *Ornithonyssus sylviarum* was also documented in Hungary, when low level of infestation of house sparrows (*Passer domesticus*) was observed in a rural area near the eastern border of the country [18]. However, the National Association of Pigeon and Small Animal Breeders of Hungary has recently experienced an increasing number of cases of massive mite infestation among racing pigeons (K. Berta, personal communication). The aim of this study was to conduct a molecular–phylogenetic analysis of mites collected from racing pigeons and human dwellings in urban areas in Hungary.

## Methods

### Samples

**Fig. 1:**
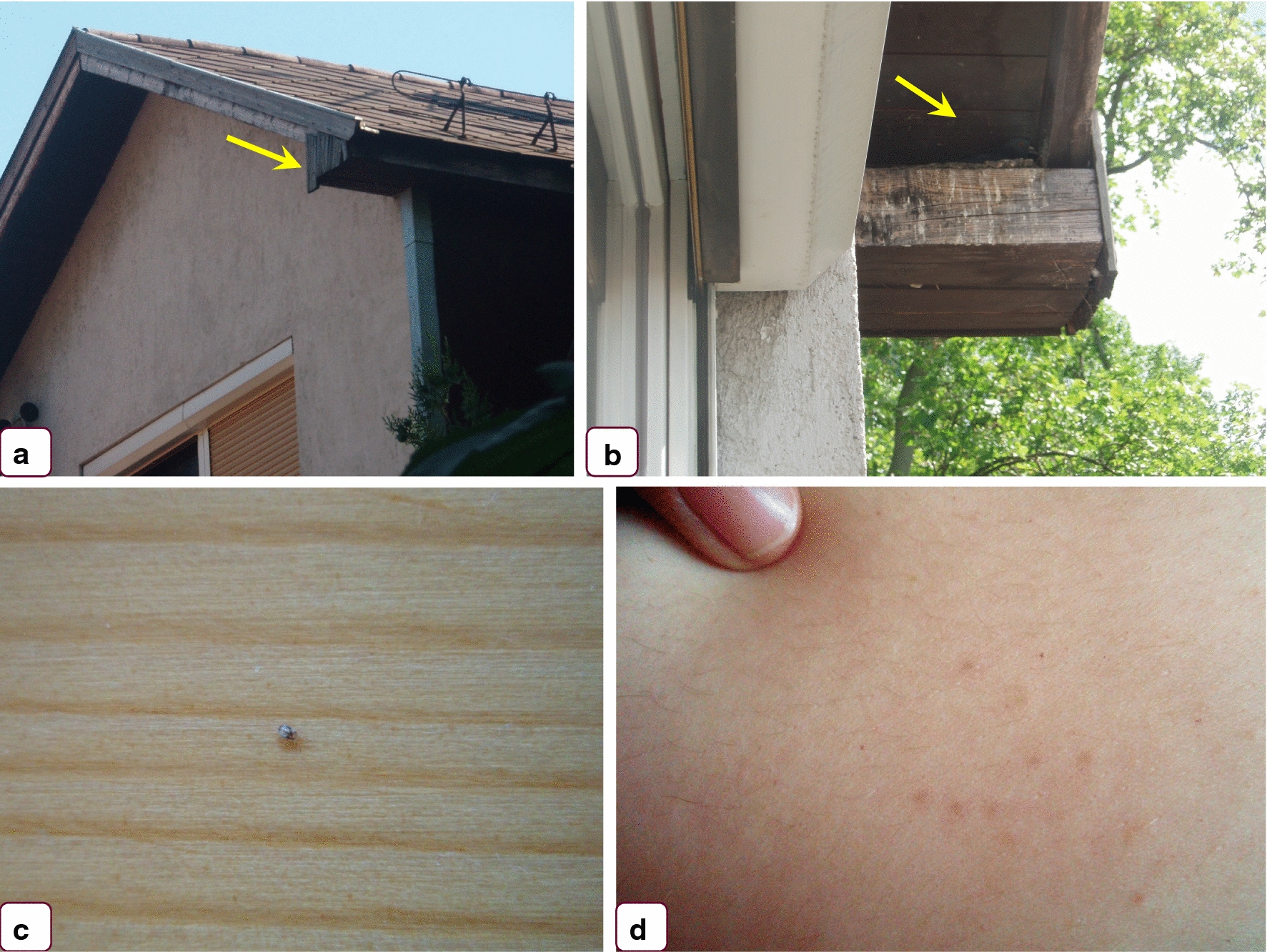
Circumstances of the first finding of *Dermanyssus gallinae* lineage L1 in Hungary (case 1). **a** The window and roof of the house, **b** the pigeon and its nest under the eaves of the roof, above the window, **c** mites entered the room and crawled over the wooden surfaces, **d** erythematous skin lesions caused by mites on the belly of the person sleeping in the room

The following specimens were used in this study:

1. Mites collected in 2012 (June) from a home in the third district of Budapest, which had an occupied pigeon (*Columba livia*) nest attached to the outside wall under the eaves. Mites invaded during the night through the window. The young couple living in the house were suffering from gamasoidosis (Fig. 1)
2. Mites collected in 2018 (August) from a home in the first district of Budapest, which had a house martin (*Delichon urbicum*) nest above the front door. Mites had invaded the home. A young, pregnant woman was present, but there was no evidence of bites.
3. Mites collected from racing pigeons in 2020 (March) in the third district of Budapest (north Hungary) and in Sellye (southern Hungary), respectively.

In each of the above cases up to 20 mites were collected and preserved in 96% ethanol. Mite species were identified morphologically according to standard keys [19–21] after clearance in lactic acid. *Dermanyssus gallinae* lineage L1 was identified molecularly. Detailed morphologic analyses (measurements of diagnostically important structures) were performed on its females (*n* = 4).

### DNA extraction

Depending on their size, up to three mites were used for DNA extraction, taking into account previous studies in which three locally collected specimens had identical cytochrome *c* oxidase subunit I (*cox*1) sequences [22], one to five mites were sequenced per isolate [8] or pools of ten mites were used for sequencing *cox*1 [10]. Prior to DNA extraction, mites were taken out from the ethanol and dried, followed by manual crushing against a sterile glass slide and transfer into Eppendorf tubes (containing tissue lysis buffer) with the aid of a pipette tip. DNA was extracted with the QIAamp DNA Mini Kit (Qiagen, Hilden, Germany) according to the manufacturer's instruction, with an overnight digestion including Proteinase K at 56 °C.

In addition, a former Hungarian sample of *D. gallinae* (*s.s.*) of unknown origin was included in the 28S rRNA gene (*28S*) analysis, as well as *Echinolaelaps echidninus* and *Laelaps hilaris* collected from a mouse and two rats in Malta (August 2017).

### Molecular and phylogenetic analyses

In order to achieve phylogenetic trees at both species- and genus/family-level resolution, an approximately 710-bp part of the *cox*1 gene and a 930-bp fragment of the *28S* gene were amplified with the primer pairs HCO2198 (5ʹ-TAA ACT TCA GGG TGA CCA AAA AAT CA-3ʹ)/LCO1490 (5ʹ-GGT CAA CAA ATC ATA AAG ATA TTG G-3ʹ), and 43F (5ʹ-GCT GCG AGT GAA CTG GAA TCA AGC CT-3ʹ)/929R (5ʹ-AGG TCA CCA TCT TTC GGG TC-3ʹ), respectively, as reported previously [1, 23, 24].

Purification and sequencing of the PCR products were performed by a commercial laboratory (Biomi Ltd., Gödöllő, Hungary). New sequences were submitted to GenBank, under accession numbers MT812940–MT812943 (*cox*1), MT813463-MT813469 (*28S*). Sequences (retrieved from GenBank) were only included in the phylogenetic analyses if they had nearly 100% coverage (i.e. aligning with a similar length and similar starting position) compared to the sequences from this study. This dataset was resampled 1000 times to generate bootstrap values. Phylogenetic analyses of *cox*1 and *28S* rRNA sequences were conducted with the maximum likelihood method (GTR model) and neighbor-joining method (p-distance model), respectively, by using the software program MEGA 7.0.

## Results

### Morphologic identification of mite species

Specimens from case 1 (associated with nesting pigeons) were identified as *D. gallinae*, whereas those from case 2 (associated with nesting house martins) and cases 3 and 4 (associated with racing pigeons) were identified as *O. sylviarum*. Within *D. gallinae*, special lineage L1 was identified molecularly (see following sections).

### Detailed morphologic analysis of *D. gallinae* lineage L1 (females)

The following are approximate measurements. The dorsal shield is 920–940 μm long, 360–370 μm wide at the apical end of the peritreme, and 290–330 μm wide at the level of coxae IV. The dorsal shield bears 15 pairs of needle-like setae (20–28 μm long), and the apical part of the dorsal shield is covered by a fine reticulate sculptural pattern. Eighteen pairs of longer (55–62 μm), needle-like setae are situated on the membranous cuticle around the dorsal shield. The sternal shield is quadrangular (70–75 μm long and 180–195 μm wide), has a fine reticulate sculptural pattern and bears two pairs of needle-like setae (50–65 μm long). Two other pairs of sternal setae, situated on the membranous cuticle posterior to the sternal shield, are longer (90–95 μm). The genital shield is linguiform, bearing one pair of pores and a pair of genital setae, its surface smooth. The anal shield is triangular (160–165 μm wide on the anterior margin and 170–175 μm long), the anterior margin is straight and the surface without a sculptural pattern. The anal opening is oval, 40–45 μm long and 30–33 μm wide. The adanal setae are 37–40 μm long and needle-like.

**Fig. 2:**
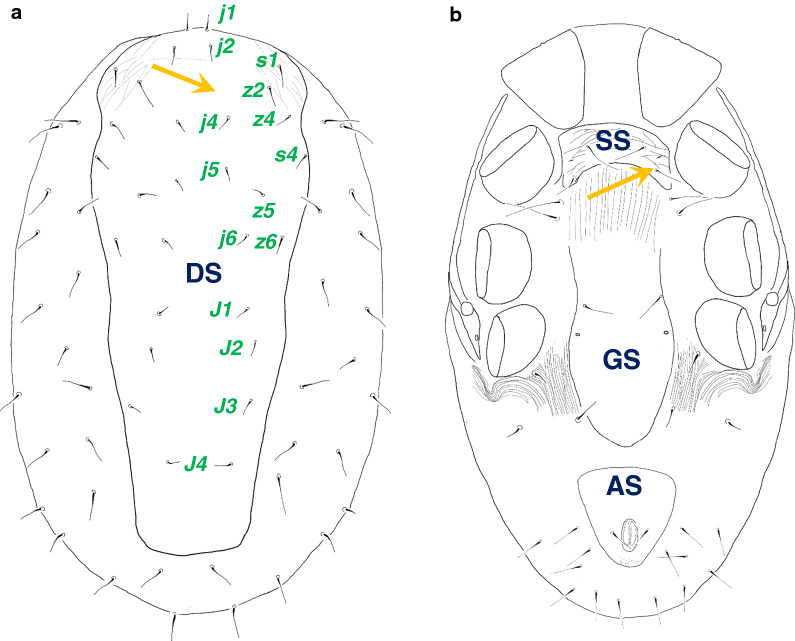
Drawings of *Dermanyssus gallinae* lineage L1 collected in Hungary: **a** dorsal view, **b** ventral view. *AS *anal shield, *DS* dorsal shield, *GS* genital shield, *SS* sternal shield. Diagnostic characters (according to [25]) are indicated with an arrow: **a** absence of *j3* setae, **b** broad sternal shield with two pairs of setae

Setae around the anal shield are needle-like and 47–54 μm long; three pairs of needle-like setae lateral to the genital shield are shorter (35–40 μm). The membranous cuticle forms a U-shaped bend posterior to coxae IV. Peritreme extends to the posterior margin of coxae I (Fig. 2).

### Molecular and phylogenetic analyses of *D. gallinae* lineage L1

The *cox*1 sequence of *D. gallinae* lineage L1 associated with pigeons in Hungary had the highest (95–99%) identity (but only 48–66% coverage) with reported sequences of this lineage from France (HQ842566, FM208734, FM208712), Italy (LT714694) and the USA (HQ842554). However, in comparison with *D. gallinae *(*s.s.*), it had only 566–568 bp of 629 bp (90–90.3%) identity with sequences reported from Israel (e.g. MH983858, MH983816) and 567 bp of 629 bp (90.1%) identity with sequences from Croatia (MH983735) and South Korea (MN648058). The* 28S* rRNA sequence of *D. gallinae* lineage L1 had the highest, i.e. 838 bp of 840 bp (99.8%), sequence identity to a corresponding sequence of *D. gallinae *(*s.s.*) (FJ911771).

**Fig. 3:**
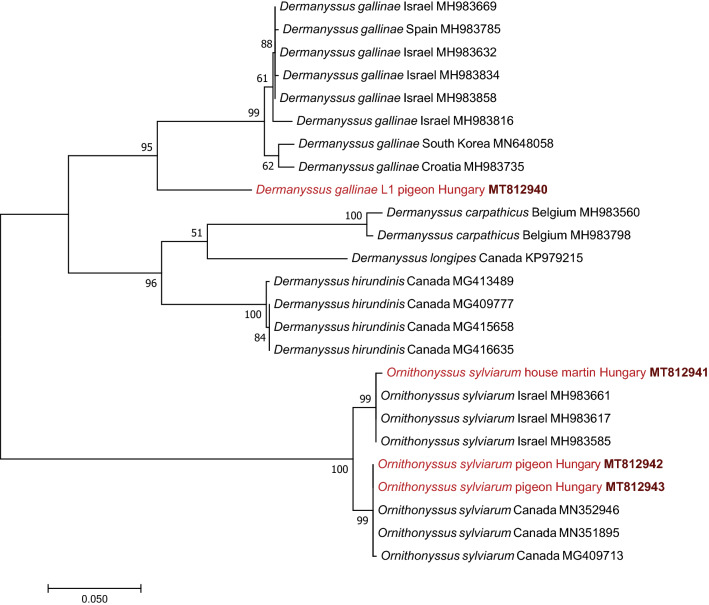
Phylogenetic tree of Dermanyssidae and Macronyssidae based on the cytochrome* c* oxidase subunit I gene (*cox*1). The tree was generated with the maximum likelihood method and general time reversible (GTR) model using the software program MEGA 7.0. Sequences obtained in this study are indicated in red, with the accession number in bold type. There were 618 positions in the final dataset. Branch lengths represent the number of substitutions per site inferred according to the scale shown

**Fig. 4:**
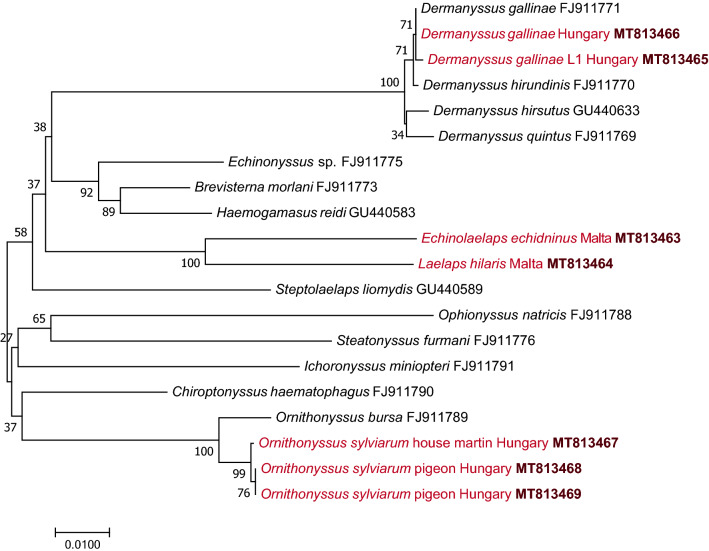
Phylogenetic tree of Dermanyssoidea based on the 28S rRNA gene (*28S*). The tree was generated with the neighbor-joining method and the p-distance model using the software program MEGA 7.0. Sequences obtained in this study are indicated in red, with the accession number in bold type. There were 775 positions in the final dataset. Branch lengths represent the number of substitutions per site inferred according to the scale shown

These differences between *D. gallinae* lineage L1 and *D. gallinae* (*s.s.*) were well-reflected by the topology of the *cox*1 phylogenetic tree, because their separation received high (95%) bootstrap support (Fig. 3). To some extent, this also applies to the low-resolution* 28S* rRNA tree, as *D. gallinae* lineage L1 had a similar degree of support for its separation (and evolutionary distance) from *D. gallinae *(*s.s.*) (FJ911771, MT813466) as did the latter from *D. hirundinis* (FJ911770) (Fig. 4).

### Molecular and phylogenetic analyses of *O. sylviarum*

The *cox*1 sequence of *O. sylviarum* associated with house martins in Hungary had the highest (99.7%; 626 of 628 bp) sequence identity with *O. sylviarum* sequences from Israel (MH983585, MH983617, MH983661; from poultry litter), while it had only 97.1% (610 of 628 bp) identity with conspecific sequences from Canada (e.g. MN351895, from a bird nest). On the contrary, two identical *cox*1 sequences of *O. sylviarum* collected from pigeons in two distant locations in Hungary showed the highest (100%; 628 of 628 bp) sequence identity with *O. sylviarum* sequences from Canada (e.g. MN351895), but considerably lower (97.5%; 612 of 628 bp) identity with the above sequences of *O. sylviarum* from Israel. These comparisons imply that there was a significant (2.9%; 18 of 628 bp) intraspecific sequence divergence between *O. sylviarum* collected from different avian host species in Hungary.

These results were confirmed with phylogenetic analyses. In the *cox*1 phylogenetic tree the separation of two divergent Hungarian *O. sylviarum* lineages, associated with house martins or pigeons, was highly supported (with 100% bootstrap), and these clustered together with sequences from Israel and Canada, respectively (Fig. 3). Their separation in the 28S rRNA gene phylogenetic analysis also received strong (99%) support (Fig. 4).

## Discussion

Human infestation with dermanyssoid mites or avian mite dermatitis (“gamasoidosis”) has not been previously reported in Hungary [7]. We report here the first case of a molecularly confirmed and phylogenetically analyzed *O. sylviarum* infestation of birds in Hungary that implicates urban environments and involves distant locations in Hungary. This is also the first account of the molecularly confirmed occurrence of *D. gallinae* lineage L1 in central Europe, because this genetic lineage has only been reported previously in southern and northern Europe (Italy [22], UK [25]).

In the present study, significant sequence divergence was found between *D. gallinae *(*s.s.*) and *D. gallinae* special lineage L1 (i.e. approximately 10%), which is well within the range of 8–18% difference in the *cox*1 sequence that separates *Dermanyssus* species [26]. This separation was also confirmed with phylogenetic analyses of two genetic markers. However, detailed morphological examination and comparison of the Hungarian pigeon-derived *D. gallinae* lineage L1 with *D. gallinae *(*s.s.*) [19, 27] revealed no major taxonomic key characters (on the basis of which these two could be consistently distinguished), thus confirming it as a cryptic species [22, 26]. At the same time, while major characters of the specimens investigated here are the same as those previously presented in the literature, the sculptural patterns of the female genital shield and the anal shield are very weakly developed, and their reticulation is hardly visible.

Based on molecular evidence and aspects of the life-cycle of *D. gallinae*, animal transportation (and not bird migration) is thought to be responsible for the dispersal of this mite species in Europe [16]. As reflected by the present results, this might also be relevant to its L1 lineage that is only associated with pigeons [22, 28], taking into account that domestic pigeons do not usually migrate and that European countries reporting the emergence (or first case) of this genetic variant show a mosaic-like distribution pattern (i.e. emergence appears to be random, not related). However, it should not be forgotten that there had been pigeon mite-associated human gamasoidosis cases prior to the molecular era (e.g. UK: [29]; Austria: [30]), which might have been caused by this lineage, indicating its long history (non-emergence).

It is well-documented that house martins in Hungary migrate to and from Africa* via* the eastern Mediterranean region, including Israel [31]. Thus, the very close molecular–phylogenetic relationship between the Hungarian and Israeli isolates of *O. sylviarum*, as demonstrated here, strongly supports our hypothesis that (at least in Hungary) long-distance dispersal and “synanthropic arrival” should be highly relevant in the case of the permanently parasitic *O. sylviarum via* natural bird migration (as opposed to *D. gallinae*, with human transportation playing a role in its dispersal events). Moreover, actively migrating house martin populations use communal roosting sites along their migratory route where birds belonging to distant populations may mix [32]. Birds spending the night in these roosts are in close proximity, usually in reed beds, where they are adjacent to each other on the same reed stem [33]. This behavior may facilitate ectoparasite transfer between individual hosts.

Surprisingly, the *cox*1 sequence of *O. sylviarum* collected from pigeons in Hungary had 100% identity to that of mites recently reported from Canada ([34]: collected in Ontario during 2015–2016 from a bird nest). Pigeons in North America are known to harbor *O. sylviarum*, but at a much lower frequency than house sparrows [35]. These results suggest a relatively recent introduction event or connection between birds of Hungarian (European) and Canadian (North American) origin. Taking into account that in the present study two pigeon farms in Hungary separated by a distance of > 200 km were found to have this haplotype, it is possible that intercontinental pigeon exhibitions, pigeon transportation and/or animal trading play a potential role in the translocation of *O. sylviarum*, similar to what has been reported for *D. gallinae* [16]. These results are in contrast to findings reported in other countries of Europe, where *O. sylviarum* specimens were shown to have remarkable sequence divergence and phylogenetic differences from North American isolates [10]. For example, the sequence divergence between French and North American isolates of *O. sylviarum* was in the range of 2–3% [8]. This is in contrast to the absence of difference (complete identity) detected in this study in a similar geographical (Palearctic *vs* Nearctic) context.

At first glance, the sequence/phylogenetic differences between *O. sylviarum* collected near the house martin nest and specimens removed from racing pigeons in Hungary could also be explained by the host-specificity of haplotypes. However, the 99.7% genetic similarity shown here between *O. sylviarum* associated with house martins in Hungary and poultry litter in Israel argues against this.

In summary, our findings highlight the importance of future large-scale molecular–phylogenetic analyses of *O. sylviarum* in an international context, especially since relevant data have been reported to date only from a few European countries.

## Conclusions

This is the first report of *D. gallinae* lineage L1 in central Europe. Detailed morphological and molecular-phylogenetic analyses of *D. gallinae* lineage L1 confirmed its status as a cryptic species within *D. gallinae* (*s.l.*). This is also the first molecularly confirmed and phylogenetically analyzed urban case of *O. sylviarum* infestation of birds in Hungary. Taking into account the well-documented latitudinal migratory routes of house martins between Hungary and Africa, *O. sylviarum* associated with this bird species most likely arrived on its host from the eastern Mediterranean region. In contrast, mites collected from pigeons in Hungary showed *cox*1 genetic homogeneity with North American *O. sylviarum*, which can only be explained by a long-distance connection of birds and their mites as part of human activity (e.g. transportation to exhibitions or trading).

## Data Availability

The sequences obtained and/or analyzed during the current study are deposited in GenBank (*cox*1 gene: MT812940-MT812943; for the* 28S* gene: MT813463-MT813469). All other relevant data are included in the manuscript and the references or are available upon request by the corresponding author.

## Abbreviations

Cox1: Cytochrome c oxidase subunit I
DS: Dorsal shield
SS: Sternal shield
GVS: Genitoventral shield
AS: Anal shield
